# Evaluation of the Quality of Guinea Fowl (*Numida meleagris*) Eggs from Free-Range Farming Depending on the Storage Period and Age of Laying Hens

**DOI:** 10.3390/foods13132161

**Published:** 2024-07-08

**Authors:** Mateusz Bucław, Michalina Adaszyńska-Skwirzyńska, Danuta Majewska, Danuta Szczerbińska, Małgorzata Dzięcioł

**Affiliations:** 1 Department of Monogastric Animal Sciences, Faculty of Biotechnology and Animal Husbandry, West Pomeranian University of Technology in Szczecin, Janickiego Str. 29, 71-270 Szczecin, Poland; mateusz.buclaw@zut.edu.pl (M.B.); danuta.majewska@zut.edu.pl (D.M.); danuta.szczerbinska@zut.edu.pl (D.S.); 2 Department of Chemical Organic Technology and Polymeric Materials, Faculty of Chemical Technology and Engineering, West Pomeranian University of Technology in Szczecin, Piastów Ave. 42, 71-065 Szczecin, Poland; malgorzata.dzieciol@zut.edu.pl

**Keywords:** guinea fowl, eggs quality, free range

## Abstract

The aim of the study was to determine the changes occurring in the eggs of helmeted guinea fowl (*Numida meleagris*) from free-range farming in relation to the laying season and storage time. The experimental material consisted of 360 guinea fowl eggs, collected in the first, second and third laying seasons and stored for 7, 14 and 21 days. After each period, physical and physicochemical characteristics of the eggs were determined, as well as the basic chemical composition and mineral content of the albumen and yolk and the yolk fatty acid profile. The age of the guinea fowls affected certain physical parameters of the eggs. The egg weight, shape index and shell thickness increased with the age of the laying hens; however, a decrease in the proportion of shell in the egg was demonstrated. Storage time had a significant effect on egg weight, weight loss during storage and air cell height. Significant differences were found in the chemical composition of guinea fowl eggs depending on the age of the laying hens. Eggs obtained from older laying hens were characterized by higher yolk fat content and lower ash content, while the albumen contained higher water content and lower ash content. During the three-year laying period, changes were observed in the mineral composition of the eggs. The fatty acid profile underwent significant changes; however, no important differences were observed in the total content of SFA, MUFA, PUFA and n-6 fatty acids. Conversely, significant differences were found for n-3 acids and the n-6/n-3 ratio. Eggs in the first and second laying seasons exhibited the most favorable composition. The slow dynamics of changes occurring in successive laying seasons and egg storage time indicated that the raw material studied was safe and could be used by consumers

## 1. Introduction

There is a preconception among present-day consumers that alternative farming systems provide greater animal welfare and, consequently, healthier and safer products compared to conventional rearing [1,2,3]. Hence, increasing attention is being paid to husbandry methods to satisfy consumer expectations regarding the quality of the final product, as well as the treatment of animals. Egg buying preferences have changed significantly over the past two decades. Currently, consumers are willing to pay more for eggs from hens kept in alternative systems, especially those from free-range farming [4]. This stems from the belief in their better sensory and nutritional quality, as well as from the increasing importance of animal welfare for the modern consumer [5,6,7].

The economic significance of chickens and the large consumption of chicken eggs have led research efforts to primarily focus on evaluating the quality concerning various husbandry systems of hens of this species. Due to the fact that hens are typically kept for one laying season, studies have often been limited to analyzing a single laying period. These works have shown that husbandry systems, including free range, affect the quality characteristics of animal products [1,8,9,10,11,12,13]. Free-range eggs were found to be richer in n-3 fatty acids, minerals, carotenoids and tocopherol [14], and they were characterized by a lower total cholesterol level and a better ratio of n-6 to n-3 fatty acids [1,11]. In addition, hens with access to green runs laid eggs with a more intense yolk color [14]. As already mentioned, chicken eggs are the most popular in the world for both direct consumption and processing. However, in some parts of the world, there is growing consumer interest in eggs from other poultry species. In Africa, guinea fowl and ostrich eggs are very popular, while in the Far East, quail and duck eggs are favored [15,16]. In West African countries, guinea fowl are the second most common source of meat and eggs after chickens. In Europe, guinea fowl farming is most significant in France, Italy, Belgium and the Scandinavian countries [15]. Guinea fowl can be raised using both intensive and alternative farming systems. Due to their ease of rearing, high resilience to environmental conditions and ability to last for many years, guinea fowls are well-suited for semi-intensive farming. In Poland, they are mainly kept in the free-range system, for 2–3 laying seasons [17]. The increasing costs of guinea fowl rearing, mainly due to rising feed prices and electricity, make single-season utilization of laying hens less profitable. Thus, extending their use to two or more production periods is advisable [16]. According to Sokołowicz and Krawczyk [18], this results in spreading the rearing costs over a higher number of eggs laid during three laying periods, contributing to increased profitability of production. Guinea fowl eggs are considered a delicacy, with very good taste qualities [15,19,20,21]. Such food products are increasingly sought after in the food market by consumers who have grown tired of chicken eggs as a regular part of their daily diet. Guinea fowls may also gain popularity due to their natural way of rearing, which aligns with the expectations of modern consumers. A literature review on guinea fowl utilization has revealed a lack of comprehensive research results, particularly regarding the long-term maintenance of these birds. It is also unknown how the quality of eggs changes in subsequent laying seasons and how the free-range system affects their physical characteristics and chemical composition. Considering that information on the nutritional value of eggs is important for consumers, it was decided to evaluate the quality of eggs of guinea fowls kept in the free-range system, taking into account their morphological structure and chemical composition in three consecutive reproductive seasons and different storage periods. The aim of this study was comprehensive research on the influence of storage time and laying season on the quality of guinea fowl eggs.

## 2. Materials and Methods

### 2.1. Research Material

The research material consisted of eggs from guinea fowl (*Numida meleagris*) raised in a free-range system. The experimental flock consisted of 35 female birds. Guinea fowls were fed with a complete pelleted feed mixture based on corn, wheat, soybean meal and barley (ME: 11.9 MJ/kg, total protein: 17%, fiber: up to 6%), formulated for this poultry species, considering the laying period (tables with the feed mixture composition are available in the Supplementary Materials). The birds had ad libitum access to feed and drinking water. The study was conducted during the first three laying seasons. The research was carried out in 2020–2022. Guinea fowl started the laying season at the turn of March/April, while the laying season ended in September. Eggs were collected for one week in each laying season in early July and stored at room temperature (20.0 ± 2.0 °C) until analysis. For analysis in each laying season, 120 eggs weighing between 42–48 g were selected, from which 40 eggs were randomly selected for each storage period (7, 14, 21 days). Selected physicochemical parameters, basic chemical composition, mineral content and fatty acid profile were determined in these samples.

### 2.2. Determination of Physicochemical Parameters

The weight of the eggs and their individual components (shell, albumen, yolk) were measured using a precise WPS 600/C/1 digital balance (Radwag, Radom, Poland), with an accuracy of 0.01 g. Weight loss during storage was determined using the same balance by subtracting the egg weight on the day of analysis from the egg weight on the day of laying. The length and width of the eggs, as well as the area and height of the thick albumen, and the height and diameter of the yolk, were determined using a 31C628 digital caliper (TOPEX, Warsaw, Poland), with an accuracy of 0.02 mm. The shape index (SI) was calculated according to the formula: SI = (egg width/egg length) × 100%. The height of the air cell was determined using a special mm scale for measuring the depth of the air cell, after outlining the air cell under an ovoscope. Shell thickness was measured using a YT-72305 micrometer with a digital display (YATO, Wrocław, Poland), with an accuracy of 0.002 mm. The albumen index (AI) was calculated using the following formula: PI = height of thick albumen/average width of thick albumen. The yolk index (YI) was calculated using the formula: yolk height/yolk diameter. Albumen and yolk pH were determined using a SG2 pH meter (Mettler-Toledo AG, Schwerzenbach, Switzerland). Yolk color was determined using the 16-point La Roche scale.

### 2.3. Determination of Basic Chemical Composition

The basic chemical composition of the eggs was determined using conventional methods. Water content was determined using a gravimetric method with an Entris 224I-1S analytical balance (Sartorius, Goettingen, Germany) and a DRY-Line 115 laboratory dryer (VWR, Quito, Ecuador). Protein content was determined via the Kjeldahl method using a KjelFlex K-360 distillation apparatus (Büchi, Flavil, Switzerland) after prior sample mineralization in a K-435 Digestion Unit mineralization apparatus (Büchi, Flavil, Switzerland). Fat content was determined using a gravimetric method with an Entris 224I-1S analytical balance (Sartorius, Goettingen, Germany) and a DRY-Line 115 laboratory dryer (VWR, Quito, Ecuador). The Soxhlet method was used for fat extraction. For this purpose, 3.0 g of yolk was weighed into a weighing vessel with an accuracy of 0.0001 g and dried in a laboratory dryer at 105 °C to constant weight. The sample was then quantitatively transferred to the extraction thimble. The thimble was weighed with an accuracy of 0.0001 g and then placed in a Soxhlet apparatus. Extraction was carried out for 12 h using diethyl ether (Chempur, Piekary Śląskie, Poland) as a solvent. After extraction, the samples were dried in Petri dishes in a fume hood until the solvent evaporated completely. Next, the thimbles were dried in a laboratory dryer at a temperature of 105 °C to constant weight, then transferred to a desiccator for stabilization, and then weighed again with an accuracy of 0.0001 g. The fat content was calculated from the difference in the mass of the thimble before and after extraction. 

Ash content was determined using a gravimetric method with an Entris 224I-1S analytical balance (Sartorius, Goettingen, Germany) and a FCF 22SP laboratory furnace (CZYLOK, Jastrzebie-Zdrój, Poland).

### 2.4. Determination of Mineral Content

The content of selected mineral components (P, K, Ca, Na, Mg, Zn, Si, Fe, Cu, Ba, Sr, Mn, Al, Se, Cr, Pb, Cd) was determined using inductively coupled plasma optical emission spectrometry (ICP-OES). The measurements were conducted using an Optima 2000DV instrument (Perkin Elmer, Waltham, MA, USA) following prior mineralization in a Speedwave Xpert microwave oven (Berghof GmbH, Eningen, Germany) equipped with a continuous temperature and pressure control system. For mineralization, 700.0 mg of frozen sample was weighed and placed in the mineralization vessel. Next, 9.0 mL of 69% HNO_3_ (Supelco, Bellefonte, PA, USA) and 1.0 mL of 30% H_2_O_2_ (Supelco, Bellefonte, PA, USA) were added. The mixture was carefully stirred and left for 15 min before sealing the vessels, then mineralization was carried out. Standard solutions (0.1; 0.5; 1.0; 2.0; 5.0; 10.0; 50.0; 150.0 mg/L) of the tested elements were prepared using the TraceCert^®^ multi-element standard (Sigma-Aldrich, St. Louis, MO, USA). Yttrium (Y) solution (Merck, Darmstadt, Germany) in HNO_3_ concentration was used in the mineralizates and standard solutions as an internal standard. The basic validation parameters of the analytical method used were estimated. Mineralized solutions were diluted directly before analysis; the blank was demineralized water used for dilution, 4.0 mL of 69% HNO_3_, yttrium solution and spectral buffer—(1% Cs in 2% HCl). The measurement results were analyzed based on standard curves plotted for each of the elements. The correlation coefficient for each curve was above 0.99. The average recovery for the samples was 96–98%. All analyses were performed in triplicate. 

### 2.5. Determination of Fatty Acid Profile in Egg Yolks—GC-MS Method

The analyses were conducted according to PN-EN ISO 12966-2 [22]. The first step involved preparing the samples for analysis. For this purpose, frozen (–82 °C) samples of egg yolks weighing approximately 1.0 g were placed in screw-cap 7.5 mL vials of amber glass with a Teflon seal (Supelco, Bellefonte, PA, USA). For extraction, 5.0 mL of a mixture of chloroform (Chempur, Piekary Śląskie, Poland) and methanol (Merck, Darmstadt, Germany) in a volume ratio of 2:1 (Folch’s mixture) was added to each vial. The samples were then sealed under a stream of nitrogen 5.0 (Air-Liquide, Kraków, Poland) and vigorously shaken for 1 h using a Vibramax 100 laboratory shaker (Heidolph, Schwabach, Germany). To separate the chloroform phase from non-lipid residues, the vials were centrifuged using a Z 205A centrifuge (Hermle, Gosheim, Germany) for 20 min at 2000 RPM. The next step was lipid hydrolysis. The chloroform layer was transferred to 4.0 mL amber glass vials (Supelco, Bellefonte, PA, USA); then, the vials were sealed with Mininert^®^ Valves (Supelco, Bellefonte, PA, USA), allowing the addition of derivatizing reagents in an inert gas atmosphere. Chloroform was evaporated from the extracts with a stream of nitrogen, after which 400.0 μL of 0.5 M KOH solution in methanol was added to the dry residue and heated for 30 min in a heating block at 80 °C. The next step was esterification. After cooling to room temperature, 500.0 μL of 14% boron trifluoride (BF_3_) solution in methanol (Sigma-Aldrich, St. Louis, MO, USA) was added to the vials and incubated at 80 °C for 30 min. For more efficient extraction of fatty acids methyl esters (FAME), 1.0 mL of saturated NaCl solution and 2.0 mL of isooctane (POCH, Gliwice, Poland) were added into the cooled vials, as an extractant. The mixture was then shaken for an hour and left for 30 min to allow phase separation. The octane layer was transferred to separate vials containing approximately 0.5 g of anhydrous sodium sulfate, and then the vials were sealed under a nitrogen stream. The dried FAME extracts were placed in vials and subjected to chromatographic analysis. The fatty acid profile of the yolk samples was determined via gas chromatography coupled with a mass spectrometry (GC-MS) using a Clarus 600 gas chromatograph combined with a Clarus 600T mass spectrometer (Perkin Elmer, Waltham, MA, USA). The instrument was equipped with a TC-80 column with a length of 60 m, 0.25 mm inner diameter and a stationary phase film thickness of 0.25 μm (GL Sciences, Tokyo, Japan). For the analyses, a standard mixture of 37 fatty acids—FAME mix C4-C24 (Sigma-Aldrich, St. Louis, MO, USA)—was used. The samples were analyzed in triplicate. 

### 2.6. Statistical Analysis

The results were statistically analyzed using the Statistica 13.1 PL software package (IBM Corp.; SPSS Statistics for Windows; Version 23.0; 2016). Two-way ANOVA was used to determine if two different factors, laying season and storage time and laying season × storage time, have an effect on a measured variable. Least squares means were obtained using the Tukey test. The significance was calculated at a 5% confidence level.

## 3. Results

### 3.1. Determination of Physicochemical Parameters

Table 1 and Table 2 present a comparison of the physical and physicochemical characteristics of eggs during the three-year laying period of guinea fowls, considering different storage periods. Laying season had a significant effect (*p* < 0.05) on: egg weight, albumen weight, yolk weight, shell percentage, yolk percentage, yolk pH, albumen pH and albumen index. On the other hand, storage time significantly (*p* < 0.05) affected egg weight, albumen weight, shell percentage, albumen percentage, yolk percentage, albumen pH, yolk color, yolk index, albumen index and air cell height. Significant interactions between the laying season and egg storage time (*p* < 0.05) were observed for albumen pH, yolk height, yolk index, and yolk pH. 

### 3.2. Determination of Basic Chemical Composition

Table 3 presents the results of the basic chemical composition of guinea fowl egg albumen depending on the laying season and storage time. The water content in egg albumen varied from year to year (from 86.7 to 89.1%) and depended only on the laying season (*p* < 0.05). No significant differences were found in the protein content (11.0%), for either laying season or storage period (*p* > 0.05). The ash content significantly decreased (*p* < 0.05) with successive laying seasons, from 0.73% in the first season to 0.58% in the third season. Interactions of laying season and egg storage time (*p* < 0.05) were found for both water and ash content.

The chemical composition of egg yolks is shown in Table 4. In the three laying seasons studied, there were no significant differences in the water content in the yolk. However, significant changes were observed during storage (*p* < 0.05)—with increasing storage time, the water content also rose (47.3% to 49.0%). There were no significant differences observed between protein content and laying season or storage time (*p* > 0.05). 

The highest fat content was determined in egg yolks in the third laying season (31.6–33.3%), with an average value of 31.9% in all yolk samples tested. A tendency towards an increase in the level of this component was observed with the age of guinea fowls (*p* < 0.05). The lowest mean ash content was determined in the yolk in the second laying season, 1.66%, while the highest was in the first, 1.93% (*p* < 0.05). The ash content decreased significantly (*p* < 0.05) with storage time, from 1.8% to 1.7%, respectively. Interactions of laying season and egg storage time (*p* < 0.05) were observed only for fat content. 

### 3.3. Determination of Mineral Content

The mineral profile of egg albumen in relation to the laying season and storage time is presented in Table 5 and Table 6. The average micronutrient contents (Table 5) for the three laying seasons were as follows: 0.09–0.17 mg Zn/kg, 3.20–4.32 mg Si/kg, 0.20–0.38 mg Fe/kg, 0.22–0.27 mg Cu/kg, 0.012–0.015 mg Ba/kg, 0.12–0.16 mg Sr/kg, 0.020–0.026 mg Mn/kg, 0.15–0.27 mg Al/kg, 0.11–0.12 mg Se/kg, 0.34–0.35 mg Cr/kg, 0.059–0.06 mg Pb/kg and 0.0048–0.0054 mg Cd/kg. The average contents of macronutrients (Table 6) in the three laying seasons were as follows: 102–104 mg P/kg, 1441–1451 mg K/kg, 42.7–47.8 mg Ca/kg, 1632–1965 mg Na/kg and 114–119 mg Mg/kg. Interactions of laying season and egg storage time (*p* < 0.05) were found for the content of the following elements: Fe, Cu, Ba, Mn, Pb, Cd, P and Na.

Table 7 and Table 8 present the contents of selected elements in the egg yolks of guinea fowls according to the laying season and storage time. The research results indicated that the average contents of trace elements (Table 7) in the three laying seasons were as follows: 44.8–45.2 mg Zn/kg, 3.67–5.11 mg Si/kg, 3.12–3.19 mg Fe/kg, 2.01–2.13 mg Cu/kg, 2.97–4.03 mg Ba/kg, 1.81–2.22 mg Sr/kg, 0.83–1.0 mg Mn/kg, 0.71–0.81 mg Al/kg, 0.54–0.74 mg Se/kg, 0.36–0.37 mg Cr/kg, 0.024–0.025 mg Pb/kg and 0.0024–0.0032 mg Cd/kg. The average macronutrient contents (Table 8) were as follows: 7018–7487 mg P/kg, 1259–1302 mg K/kg, 1938–2033 mg Ca/kg, 511–1965 mg Na/kg and 140–155 mg Mg/kg. Interactions of laying season and egg storage time (*p* < 0.05) were found for the content of the following elements: Zn, Si, Ba, Se, Pb and Na.

### 3.4. Determination of Fatty Acid Profile in Egg Yolks

Table 9 summarizes the fatty acid profile of egg yolks according to laying season and storage time. Egg yolks from the three laying periods had the highest content of palmitic acid (C16:0)—11.8%, stearic acid (C18:0)—8.11%, oleic acid (C18:1 n-9c)—48.53% and linoleic acid (LA—C18:2 n-6c)—27.7%. Laying period significantly affected the content of all acids tested except palmitic acid (C16:0), oleic acid (C18:1 n-9c) and linoleic acid (C18:2 n-6c) (*p* > 0.05). Storage period affected only the content of the following acids: palmitic (C16:0), oleic (C18:1 n-9c), linoleic (LA—C18:2 n-6c), linolenic (ALA—C18:3 n-3) and eicosatrienoic (C20:3 n-3) acids. Regarding other acids, there were no statistical differences between their content and storage period. A significant (*p* ≤ 0.05) interaction between the laying season and storage time was observed for all examined acids with the exception of myristic acid (C14:0) and docosahexaenoic acid (C22:6 n-3). In addition, there was a significant (*p* ≤ 0.05) interaction of laying season and storage time for saturated fatty acids (SFA), monounsaturated fatty acids (MUFA), polyunsaturated fatty acids (PUFA), n-6 fatty acids and the ratio of unsaturated fatty acids to saturated fatty acids (UFA/SFA). Egg yolks of guinea fowls contained on average 20.3% SFA, 50.26% MUFA and 30.36% PUFA. The highest n-6/n-3 acid ratio, which differed significantly from previous seasons, was observed in the last laying season—24.6% (*p* < 0.05). No significant differences were found in the UFA/SFA ratios for the laying season (*p* > 0.05); however, variations were observed depending on the egg storage time (*p* < 0.05). 

## 4. Discussion

Guinea fowl eggs are considered a delicacy and a dietetic product due to their low cholesterol level, low calorie count and high vitamin A content [15,20]. In terms of protein content, guinea fowl eggs are distinguished by its slightly higher albumen content compared to other bird species [16]. Research indicates that the qualitative characteristics of eggs change during successive laying stages [8,23]. A number of changes affecting egg quality can also occur during their storage [24,25,26,27,28]. Egg weight, yolk and albumen weight and shell thickness are important features influencing egg quality [25]. The average weight of guinea fowl eggs from the three laying seasons was 45.5 g; only the first season showed a statistically lower weight of 43.4 g. Published reports indicate that the weight of guinea fowl eggs ranges from 38 to 45 g [16]. According to many authors [29,30,31], egg weight is significantly influenced by the age of the laying hens through its effect on increasing the proportion of yolk in the overall egg weight, while simultaneously decreasing the egg’s albumen content. Storage time and temperature is another factor affecting egg quality. It should be noted that in eggs, as in almost all biological systems, changes in one characteristic entail transformations that affect other qualitative features of the raw material. In the case of eggs, the primary change over time is a loss of their mass. This variability is continuous and linear, and importantly, it has been consistently observed regardless of changes in egg storage temperature or the use of protective substances [31,32,33]. During three weeks of storage at room temperature, the average weight loss of eggs was 0.63 g in the first laying season and 0.62 g in the second and third seasons. Water evaporation through the shell into the surrounding environment is accompanied by its internal movement from the albumen through the vitelline membrane into the yolk, resulting in an increase in its volume. The average yolk weight was 15.5 g and its value increased with each subsequent laying season. Storage time had a significant effect on yolk weight only in the first laying season. A similar relationship was confirmed for albumen weight, with an average value of 23.1 g. Meanwhile, the proportion of yolk increased with storage time, while the albumen percentage decreased. A similar trend was demonstrated in the study by Menezes et al. [34]. Guinea fowl eggs are known for having thicker and more durable shells compared to chicken eggs, which allows for longer storage periods [9]. Natural aging of an egg leads to biophysical and chemical changes in its contents [26,35]. It has been observed that alterations in egg quality are associated with temperature and storage time [25,34,36]. According to Silversides and Scott [37], the age of the laying hens also affects the quality of the eggshell, including its thickness, whose average value was 0.52 mm and increased with successive laying seasons, while its average weight and percentage were 6.90 g and 15.2%, respectively. Shell percentage was significantly affected by both laying season (*p* < 0.01) and storage time (*p* < 0.05). In contrast, Van den Brand et al. [5] found no influence of hen age on eggshell thickness but observed only a decrease in egg shape index. In the present study, the average egg shape index during three years was 76.6%. According to some researchers, the egg shape index is influenced by the egg’s weight—as the weight increases, the eggs take on an elongated shape, resulting in a lower egg shape index [35]. Another factor influencing egg quality is freshness, which is assessed based on characteristics such as pH and air cell size. The present study found that the pH value of the albumen slightly increased with storage time, while it decreased with each subsequent year; its average value was 9.28. Wilkanowska and Kokoszyński [38] observed a similar pH value of the egg albumen, i.e., 9.01. In contrast, the average pH value of the yolk was 6.14. The highest yolk pH was recorded in the third laying season—6.27. Wilkanowska and Kokoszyński [38] compared white and grey guinea fowl eggs and found significant differences between varieties—the average yolk acidity was 6.13. When evaluating the yolk color during the present three-year experiment, it was found to be consistent across all seasons of guinea fowl farming, averaging 10.0 points on a 15-point La Roche scale. A similar color was recorded by Wilkanowska and Kokoszyński [38], while more intense yolk coloration, 14.6 points, was obtained by Banaszewska et al. [36]. The differences can be explained by different diets of guinea fowls, as yolk color is related to the content of natural pigments in the feed. The average depth of the air cell during the experiment was 1.91 mm, and its value increased with longer egg storage (*p* < 0.05). No significant differences were found in the depth of the air cell between successive laying seasons (*p* > 0.05). Higher albumen height and smaller spreading area indicate better quality, a finding confirmed in our own study. Moreover, significant influence of both laying season and egg storage time on albumen height, spread and albumen index was observed (*p* < 0.05). Wilkanowska and Kokoszyński [38] demonstrated an albumen height of 4.7 mm (white guinea fowl) and 6 mm (grey guinea fowl), resulting in an average albumen index of 7.67. The average yolk index was 36% and decreased with prolonged egg storage (*p* < 0.05). The laying season did not have a significant impact on this characteristic. Wilanowska and Kokoszyński [38] reported a higher yolk index, the value of which was 38.83. 

The basic chemical composition of an egg determines its nutritional value. During the three laying seasons, the average values of the following components were determined in the albumen: water—87.5%, protein—11%, and ash—0.66%. The highest water content was found in the last laying season—89.1%. There was a significant effect of laying season on water and ash content (*p* < 0.05), while storage time significantly affected only ash percentage. In the study by Teodorescu et al. [39], the average water content in guinea fowl egg albumen was 74.6%, while the protein content was 10.5%. In the present study, the average water content in the yolk was 48.8%, the protein content was 16.6%, the fat content was 31.9% and the ash content was 1.75%. Significant differences were observed for storage time—the water content increased with storage time (from 47.3% to 49.0%), while the ash content decreased. No significant differences were found between the protein content in the yolk and laying season or storage time (*p* > 0.05). The highest fat content was determined in egg yolks in the third laying season (31.6–33.3%), with an average value of 31.9% in all yolk samples examined. An increase in the level of this component was observed with the age of guinea fowls (*p* < 0.05). A similar content of basic chemical components in the yolks of gray guinea fowl (*Numida Meleagris*) was obtained by Teodorescu et al. [39]. 

Mineral metabolism is an extremely important factor determining metabolic changes occurring in laying hens’ bodies, associated also with egg production. Metabolic disorders involving these components often occur in laying hens undergoing production intensification [40]. Due to the limited reports on the mineral composition of guinea fowl eggs, the most important components, which appear to be significant for poultry health and the quality of products obtained from them, were analyzed. The mineral composition of eggs can be modified, for example, through the selection of housing conditions and feeding [41,42,43]. Studies on chicken egg demonstrated that its content is particularly rich in ions of such elements as phosphorus, chlorine, potassium, sodium, sulfur, calcium, magnesium and iron. Ions of zinc, fluorine, bromine, iodine, copper, manganese, arsenic, boron, barium, chromium, aluminum, silicon, lithium, molybdenum, lead, rubidium, selenium, strontium, cobalt, titanium, uranium, vanadium and silver are present in smaller or even trace amounts [40]. In the mineral composition of the egg, phosphorus is the most significant in the group of macronutrients. Consumption of one medium-sized egg covers 13% of a person’s daily requirement for this element. There is also a high content of systemic electrolytes, such as sodium and chloride (covering 12% of daily requirements), with a small 2% supply of potassium. Among trace elements, chicken eggs are characterized by a high content of iron and zinc, with the consumption of one egg covering 10% and 6% of human requirement for these metals, respectively. Meanwhile, the average content of iodine and selenium can cover this requirement by 7.5% and 13%, respectively [44]. Based on the conducted research, it has been found that guinea fowl eggs are also a rich source of macro- and micronutrients. Among the minerals analyzed, albumen contained the highest amount of sodium and potassium, averaging 1781 mg Na/kg and 1455 mg K/kg, respectively (Table 6). Taking into account the contents of the elements determined in the egg albumen (Table 5 and Table 6), they can be arranged in the following order: Na > K > Mg > P > Ca > Si, followed by elements present at concentrations below 1 mg/kg: Sr > Cu > Fe > Sr > Al > Se > Zn > Pb > Mn > Cd. Laying season had a significant effect on the content of all micronutrients analyzed except for Cr and Pb (*p* > 0.05). As storage time increased, the content of Fe, Cu, Ba, Sr, Mn, Pb and Cd in egg whites changed (*p* < 0.05). There was no significant effect of storage time on the content of Zn, Si, Al, Se and Cr (*p* > 0.05). Significant differences in the content of Ca and Na depending on the laying season were observed for macronutrients in the egg albumen, while storage time influenced the concentration of P, Ca and Na (*p* < 0.05). Teodorescu et al. [39] described a similar sequence of elements in the egg albumen of guinea fowl eggs. Their study only found a higher content of Fe (18.99 mg/kg) and Zn (2.71 mg/kg). On the other hand, Bashir et al. [4] determined the following content of selected mineral components in guinea fowl egg albumen (mg/kg): Na—1990; K—1195; Ca—92.3; Fe—44.5. The present study showed that the Zn content varied for each laying period. The highest zinc content was found in the egg albumen during the second laying period, averaging 0.17 mg/kg, while the lowest was observed in the third laying period. Albumen of eggs in the third laying season had the highest accumulation of Si, with an average value of 4.32 mg/kg. The lowest amount of Si was detected in the albumen of eggs in the first and second laying seasons, corresponding to 3.34 and 3.20 mg/kg, respectively. Meanwhile, the iron content varied from 0.20 to 0.38 mg/kg, depending on the laying season. The analyzed albumen contained an average of 0.27 to 0.22 mg/kg of Cu in the three laying seasons, with the highest amount recorded in the first season (0.27 mg/kg). The average Ba content was comparable in the first and second laying period (0.015 mg/kg) and slightly decreased in the third season (0.012 mg/kg). The average Sr content varied from 0.12 to 0.16 mg/kg, reaching the highest value in the third laying season. The raw material tested contained relatively small amounts of Mn, in the range of 0.020 to 0.026 mg/kg over the three laying seasons. Among the elements analyzed in the yolk, the highest average content (Table 7 and Table 8) was determined for P—7294 mg/kg, Ca—1973 mg/kg and K—1277 mg/kg. The elements analyzed in the yolk can be ranked according to their content in the following order: P > Ca > K > Na > Mg > Zn > Si > Ba > Fe > Sr > Cu, followed by elements present in concentrations below 1 mg/kg: Mn > Al > Se > Cr > Pb > Cd. With each successive laying season, the yolk of eggs exhibited changes in the content of P, Na, Mg, Si, Ba, Sr, Mn, Al, Se and Cd (*p* < 0.01). The experiment demonstrated that the concentration of Na, Mg, Zn, Ba and Se in the yolk changed with storage time (*p* < 0.05). Other authors also observed the similar contents of minerals in the yolks of guinea fowl eggs [39]. Bashir et al. [45] analyzed the content of selected mineral components in the yolks of guinea fowl eggs and found higher concentrations of Na—1910 mg/kg, K—3215 mg/kg and Fe—124.5 mg/kg but a lower concentration of Ca—266 mg/kg.

Foods of animal origin are rich in fats high in saturated fatty acids (SFA). A prerequisite for a healthy diet is food containing essential fatty acids (EFA). Dietary changes can impact the prevalence of chronic conditions such as obesity, type II diabetes, cancer, atherosclerosis, hypertension and cardiovascular diseases. These diseases result not only from low consumption of EFA but also from an improper ratio of n-6 to n-3 fatty acids [21,46]. The increase in the consumption of n-6 fatty acids in recent years is attributed to the advancement of modern technologies in food production, such as agronomy and the feed industry, involving introduction of cereal grains rich in n-6 fatty acids into animal feed. This has led to an unfavorable ratio of n-6 to n-3 fatty acids. Egg yolks are a rich source of lipids, constituting approximately 64% of their dry mass, including phospholipids, which make up one-third of the lipid fraction [47]. The composition of fatty acids contained in egg lipids, such as saturated fatty acids (SFA), monounsaturated fatty acids (MUFA) and polyunsaturated fatty acids (PUFA), is of great interest from a human health perspective. For example, insufficient intake of PUFA n-3, especially docosahexaenoic acid (DHA, C22:6 n-3), negatively affects brain growth and functional parameters in infants [48]. Long-chain fatty acids are not found in phospholipids isolated from plant sources; therefore, the phospholipids present in egg yolks may be a component of functional food. In the current study, the fatty acid profile was determined in the yolk samples collected during the analysis (Table 9). The fatty acid profile present in the lipids of the egg yolk changed during the three-year laying period. Egg yolks of guinea fowls contained on average 20.3% SFA, 50.2% MUFA and 29.4% PUFA. The results concerning the fatty acid profile content in guinea fowl eggs are varied. The literature indicates that guinea fowl egg yolks are a rich source of EFA from both the n-3 and n-6 groups, whose amount depends mainly on the genotype, age of the laying hens, origin, and nutrition [38,49,50,51]. The percentage of fatty acids determined in the present study was similar to the data presented by Polat et al. [49]. A study by Mohsenpur et al. [52] showed that the content of SFA in the yolk was 38.14%, MUFA 38.71% and PUFA 22.09%. Meanwhile, Bondoc et al. [51] reported the following fatty acid contents: 39.47% SFA, 34.47% MUFA and 17.30% PUFA. Similar PUFA profiles were demonstrated by Kouassi et al. [50]. The products of EFA metabolism from the n-3 and n-6 groups significantly affect cellular biochemical processes, yet their different chemical structure determines varying biological activity. Therefore, it is crucial to properly balance the amounts of both groups of fatty acids in the diet [46]. Achieving optimal health benefits requires the proper quantitative composition and appropriate proportions of ingested fatty acids, making it important from a nutritional standpoint to determine their relative ratio. According to literature data, the ratio of n-6 to n-3 fatty acids should be in the range 4–6:1 [53]. In the present research, the most favorable ratio was calculated for the yolks of guinea fowl eggs in the first and second laying seasons (averaging 17.5–17.7:1). Egg yolks in the third laying season showed significantly higher values of the analyzed indicator, averaging 24.6:1. In a study by Bondoc et al. [51], the n-6/n-3 fatty acids ratio in the egg yolks of guinea fowls was 31.71, while for other poultry species it was as follows: turkeys—57.06; quails—64.66; chickens—26.14. The relatively high proportion of EFA and relatively favorable n-6/n-3 ratio found in the present study indicate the high nutritional value of guinea fowl eggs. On the other hand, in a study by Oguntona and Hughes [54], oleic acid (C18:1) was the main unsaturated fatty acid in guinea fowl egg yolks, while palmitic acid (C16:0) was the main saturated fatty acid. Overall, 49% of the fatty acids belonged to SFA, while 51% were UFA. There was no significant difference (*p* > 0.05) in the fatty acid profile in eggs collected during the three laying periods (at week 8, 12 and 16). 

## 5. Conclusions

Summarizing the results obtained it can be concluded that 21 days storage time of guinea fowl eggs was characterized by small dynamics of undesirable changes. It was demonstrated that it significantly influenced only egg weight loss and air cell height during storage. On the other hand, the age of the guinea fowls affected certain physical parameters and the chemical composition of the eggs. The egg weight, shape index and shell thickness increased with the age of the laying hens. A decrease in the shell percentage was observed in successive laying seasons. Moreover, significant differences were found in the chemical composition of guinea fowl eggs depending on the age of the laying hens. Eggs obtained from older birds had a higher yolk fat content and lower ash content, while older eggs showed higher water content in the albumen and lower ash content. During the three-year laying period, alterations were observed in the mineral composition of the eggs. It should be noted that the fatty acid profile underwent significant changes; however, there were no important alterations observed in the total content of SFA, MUFA, PUFA and n-6 fatty acids. Significant differences were found for n-3 acids and the n 6/n-3 ratio, which was most favorable in the first and second laying seasons. It should be emphasized that none of the observed changes disqualify the tested guinea fowl eggs as valuable food products.

## Figures and Tables

**Table 1:** Physical characteristics of eggs during three-year laying seasons of guinea fowls.

Item	Storage Time	Laying Season (Average ± SD)			
		I	II	III	Average
Whole egg characteristics					
Egg weight (g)	7 days	44.8 ^abc^ ± 1.3	46.5 ^a^ ± 1.6	46.8 ^a^ ± 2.0	46.1 _d_ ± 1.9
	14 days	44.3 ^bc^ ± 1.3	45.8 ^a^ ± 1.7	46.1 ^a^ ± 1.6	45.5 _e_ ± 1.7
	21 days	43.4 ^c^ ± 1.5	45.1 ^ab^ ± 1.1	45.3 ^ab^ ± 1.5	44.8 _f_ ± 1.6
	Average	44.2 _b_ ± 1.4	45.8 _a_ ± 1.6	46.1 _a_ ± 1.8	45.5 ± 1.8
Egg weight loss during storage (g)	7 days	0.17 ^d^ ± 0.01	0.17 ^de^ ± 0.01	0.16 ^e^ ± 0.01	0.17 _f_ ± 0.01
	14 days	0.60 ^b^ ± 0.01	0.59 ^bc^ ± 0.01	0.59 ^c^ ± 0.01	0.59 _e_ ± 0.01
	21 days	1.11 ^a^ ± 0.01	1.10 ^a^ ± 0.01	1.10 ^a^ ± 0.01	1.10 _d_ ± 0.01
	Average	0.63 _a_ ± 0.39	0.62 _b_ ± 0.39	0.62 _b_ ± 0.39	0.62 ± 0.38
Egg shape index (%)	7 days	76.2 ^ab^ ± 2.6	76.3 ^ab^ ± 4.3	77.5 ^a^ ± 3.3	76.6 _d_ ± 3.6
	14 days	76.4 ^ab^ ± 2.9	75.9 ^ab^ ± 3.6	77.1 ^ab^ ± 2.7	76.5 _d_ ± 3.1
	21 days	76.7 ^ab^ ± 2.9	75.6 ^b^ ± 3.6	77.3 ^a^ ± 3.3	76.6 _d_ ± 3.3
	Average	76.4 _b_ ± 2.8	76.0 _b_ ± 3.9	77.3 _a_ ± 3.1	76.6 ± 3.3
Air cell height (mm)	7 days	1.18 ^c^ ± 0.33	1.12 ^c^ ± 0.36	1.17 ^c^ ± 0.36	1.16 _f_ ± 0.35
	14 days	2.07 ^b^ ± 0.63	2.13 ^b^ ± 0.35	2.10 ^b^ ± 0.37	2.10 _e_ ± 0.46
	21 days	2.55 ^a^ ± 0.37	2.58 ^a^ ± 0.38	2.51 ^a^ ± 0.53	2.54 _d_ ± 0.44
	Average	1.88 _a_ ± 0.73	1.88 _a_ ± 0.72	1.96 _a_ ± 0.72	1.91 ± 0.72
Shell characteristics					
Shell weight (g)	7 days	6.87 ^a^ ± 0.44	6.93 ^a^ ± 0.51	6.95 ^a^ ± 0.57	6.92 _d_ ± 0.51
	14 days	6.85 ^a^ ± 0.46	6.90 ^a^ ± 0.47	6.93 ^a^ ± 0.44	6.90 _d_ ± 0.44
	21 days	6.83 ^a^ ± 0.39	6.86 ^a^ ± 0.30	6.87 ^a^ ± 0.61	6.86 _d_ ± 0.49
	Average	6.85 _a_ ± 0.46	6.90 _a_ ± 0.43	6.92 _a_ ± 0.54	6.90 ± 0.48
Shell percentage (%)	7 days	15.4 ^ab^ ± 0.9	14.9 ^b^ ± 0.8	14.8 ^b^ ± 1.0	15.0 _e_ ± 0.9
	14 days	15.5 ^ab^ ± 0.8	15.1 ^ab^ ± 0.7	15.0 ^ab^ ± 0.8	15.2 _de_ ± 0.8
	21 days	15.7 ^a^ ± 0.7	15.2 ^ab^ ± 0.6	15.2 ^ab^ ± 1.0	15.3 _d_ ± 0.9
	Average	15.5 _a_ ± 0.8	15.1 _b_ ± 0.7	15.0 _b_ ± 0.9	15.2 ± 0.9
Average shell thickness (mm)	7 days	0.51 ^ab^ ± 0.04	0.53 ^ab^ ± 0.03	0.54 ^a^ ± 0.05	0.52 _d_ ± 0.04
	14 days	0.50 ^b^ ± 0.04	0.52 ^ab^ ± 0.04	0.52 ^ab^ ± 0.04	0.51 _d_ ± 0.04
	21 days	0.50 ^b^ ± 0.02	0.52 ^ab^ ± 0.05	0.53 ^ab^ ± 0.06	0.51 _d_ ± 0.04
	Average	0.50 _b_ ± 0.03	0.52 _a_ ± 0.04	0.53 _a_ ±0.05	0.52 ± 0.04
Main Effect (*p*-Value)					
	Laying Season (L)		Storage Time (ST)	L × ST	
Egg weight	<0.0001		<0.0001	0.9943	
Egg weight loss during storage	<0.0001		<0.0001	0.7731	
Egg shape index	<0.0001		0.8213	0.4274	
Air cell height	0.9760		<0.0001	0.9281	
Shell weight	0.6730		0.7213	0.9996	
Shell percentage	0.0003		0.0467	0.9939	
Average shell thickness	0.0001		0.1011	0.8205	

_a,b_—Statistically significant differences (*p* ≤ 0.05) for the laying season are marked with different letters in the subscript; _d–f_—statistically significant differences (*p* ≤ 0.05) for the storage time are marked with different letters in the subscript; ^a–e^—statistically significant differences (*p* ≤ 0.05) for interaction type of laying season × storage time are marked with different letters in the superscript.

**Table 2:** Physical characteristics of egg content during three-year laying seasons of guinea fowls.

Item	Storage Time	Laying Season (Average ± SD)			
		I	II	III	Average
Albumen characteristics					
Albumen weight (g)	7 days	23.3 ^abc^ ± 1.1	24.0 ^ab^ ± 1.3	24.1 ^a^ ± 1.3	23.8 _d_ ± 1.3
	14 days	22.5 ^cd^ ± 0.8	23.3 ^abc^ ± 0.8	23.3 ^bc^ ± 1.3	23.1 _e_ ± 1.1
	21 days	21.6 ^d^ ± 1.0	22.5 ^cd^ ± 0.8	22.4 ^cd^ ± 0.9	22.2 _f_ ± 1.0
	Average	22.5 _b_ ± 1.2	23.3 _a_ ± 1.2	23.3 _a_ ± 1.3	23.1 ± 1.3
Albumen percentage (%)	7 days	52.0 ^a^ ± 2.1	51.7 ^ab^ ± 1.7	51.3 ^ab^ ± 1.5	51.6 _d_ ± 1.8
	14 days	50.9 ^abc^ ± 1.3	50.9 ^abc^ ± 1.1	50.4 ^bc^ ± 1.6	50.7 _e_ ± 1.4
	21 days	49.8 ^c^ ± 1.3	49.8 ^c^ ± 1.0	49.5 ^c^ ± 1.5	49.7 _f_ ± 1.3
	Average	51.0 _a_ ± 1.9	50.8 _a_ ± 1.5	50.4 _a_ ± 1.7	50.7 ± 1.7
Thick albumen index (%)	7 days	0.073 ^bc^ ± 0.013	0.086 ^a^ ± 0.015	0.080 ^ab^ ± 0.017	0.079 _d_ ± 0.016
	14 days	0.055 ^de^ ± 0.012	0.069 ^bc^ ± 0.014	0.061 ^cd^ ± 0.013	0.062 _e_ ± 0.014
	21 days	0.043 ^e^ ± 0.009	0.050 ^de^ ± 0.014	0.048 ^e^ ± 0.011	0.047 _f_ ± 0.011
	Average	0.059 _b_ ± 0.017	0.069 _a_ ± 0.020	0.063 _b_ ± 0.019	0.064 ± 0.019
Albumen pH	7 days	9.41 ^bc^ ± 0.10	9.06 ^de^ ± 0.17	9.02 ^e^ ± 0.21	9.14 _f_ ± 0.25
	14 days	9.46 ^ab^ ± 0.05	9.32 ^c^ ± 0.06	9.50 ^ab^ ± 0.14	9.44 _d_ ± 0.13
	21 days	9.55 ^a^ ± 0.08	9.31 ^c^ ± 0.13	9.13 ^d^ ± 0.08	9.27 _e_ ± 0.20
	Average	9.47 _a_ ± 0.10	9.22 _b_ ± 0.18	9.20 _b_ ± 0.25	9.28 ± 0.23
Yolk characteristics					
Yolk weight (g)	7 days	14.6 ^d^ ± 0.9	15.6 ^abc^ ± 0.7	15.8 ^a^ ± 0.9	15.4 _d_ ± 1.0
	14 days	14.9 ^cd^ ± 0.7	15.6 ^abc^ ± 0.7	15.9 ^a^ ± 0.7	15.6 _d_ ± 0.8
	21 days	15.0 ^bcd^ ± 0.8	15.7 ^abc^ ± 0.6	16.0 ^a^ ± 0.8	15.7 _d_ ± 0.8
	Average	14.8 _b_ ± 0.8	15.6 _a_ ± 0.7	15.9 _a_ ± 0.8	15.5 ± 0.9
Yolk percentage (%)	7 days	32.6 ^d^ ± 1.9	33.5 ^cd^ ± 1.6	33.8 ^bcd^ ± 1.5	33.4 _f_ ± 1.7
	14 days	33.7 ^bcd^ ± 1.4	34.1 ^abcd^ ± 0.9	34.5 ^abc^ ± 1.6	34.2 _e_ ± 1.4
	21 days	34.5 ^abc^ ± 1.6	34.9 ^ab^ ± 1.0	35.3 ^a^ ± 1.4	35.0 _d_ ± 1.4
	Average	33.5 _b_ ± 1.8	34.2 _ab_ ± 1.4	34.6 _a_ ± 1.6	34.2 ± 1.7
Yolk index (%)	7 days	40.0 ^a^ ± 0.04	39.0 ^ab^ ± 0.03	40.0 ^a^ ± 0.03	40.0 _d_ ± 0.03
	14 days	37.0 ^bc^ ± 0.04	36.0 ^b^ ± 0.03	36.0 ^b^ ± 0.03	36.0 _e_ ± 0.04
	21 days	31.0 ^c^ ± 0.04	30.0 ^c^ ± 0.01	33.0 ^c^ ± 0.03	32.0 _f_ ± 0.03
	Average	37.0 _a_ ± 0.06	36.0 _a_ ± 0.04	36.0 _a_ ± 0.04	36.0 ± 0.05
Yolk pH	7 days	5.90 ^c^ ± 0.18	6.05 ^bc^ ± 0.10	6.53 ^a^ ± 0.20	6.21 _d_ ± 0.33
	14 days	6.06 ^bc^ ± 0.28	6.04 ^bc^ ± 0.22	6.12 ^b^ ± 0.28	6.08 _e_ ± 0.26
	21 days	6.13 ^bc^ ± 0.16	6.09 ^bc^ ± 0.18	6.09 ^bc^ ± 0.34	6.10 _de_ ± 0.26
	Average	6.02 _b_ ± 0.23	6.06 _b_ ± 0.17	6.27 _a_ ± 0.34	6.14 ± 0.30
Yolk color (pkt)	7 days	10.4 ^a^ ± 0.9	10.4 ^a^ ± 0.8	10.3 ^a^ ± 0.9	10.3 _d_ ± 0.8
	14 days	10.1 ^a^ ± 1.0	10.0 ^a^ ± 0.7	10.0 ^a^ ± 0.9	10.0 _d_ ± 0.9
	21 days	9.76 ^a^ ± 0.83	9.65 ^a^ ± 0.75	9.67 ^a^ ± 0.87	9.68 _e_ ± 0.82
	Average	10.1 _a_ ± 0.9	10.0 _a_ ± 0.8	10.0 _a_ ± 0.9	10.0 ± 0.9
Main Effect (*p*-Value)					
	Laying Season (L)		Storage Time (ST)	L × ST	
Albumen weight	<0.0001		<0.0001	0.9991	
Albumen percentage	0.1128		<0.0001	0.9384	
Thick albumen index	<0.0001		<0.0001	0.7398	
Albumen pH	<0.0001		<0.0001	<0.0001	
Yolk weight	<0.0001		0.1809	0.9257	
Yolk percentage	0.0002		<0.0001	0.9464	
Yolk index	0.1934		<0.0001	0.0451	
Yolk pH	<0.0001		0.0913	<0.0001	
Yolk color	0.7910		<0.0001	0.9965	

_a,b_—Statistically significant differences (*p* ≤ 0.05) for the laying season are marked with different letters in the subscript; _d–f_—statistically significant differences (*p* ≤ 0.05) for the storage time are marked with different letters in the subscript; ^a–e^—statistically significant differences (*p* ≤ 0.05) for interaction type of laying season × storage time are marked with different letters in the superscript.

**Table 3:** Basic chemical composition of guinea fowl egg albumen.

Item	Storage Time	Laying Season (Average ± SD)			
		I	II	III	Average
Moisture (%)	7 days	87.3 ^cd^ ± 0.5	87.0 ^de^ ± 0.5	88.4 ^bc^ ± 0.9	87.5 _d_ ± 0.9
	14 days	86.7 ^de^ ± 0.5	86.7 ^de^ ± 0.7	89.6 ^a^ ± 1.1	87.6 _d_ ± 1.6
	21 days	86.2 ^de^ ± 0.8	86.0 ^e^ ± 0.7	89.5 ^ab^ ± 1.3	87.2 _d_ ± 1.9
	Average	86.7 _b_ ± 0.7	86.5 _b_ ± 0.7	89.1 _a_ ± 1.2	87.5 ± 1.5
Protein (%)	7 days	11.0 ^a^ ± 0.1	11.0 ^a^ ± 0.1	11.1 ^a^ ± 0.1	11.0 _d_ ± 0.1
	14 days	11.1 ^a^ ± 0.1	11.0 ^a^ ± 0.1	11.0 ^a^ ± 0.1	11.0 _d_ ± 0.1
	21 days	11.0 ^a^ ± 0.1	11.0 ^a^ ± 0.1	11.1 ^a^ ± 0.1	11.0 _d_ ± 0.1
	Average	11.0 _a_ ± 0.1	11.0 _a_ ± 0.1	11.1 _a_ ± 0.1	11.0 ± 0.1
Ash (%)	7 days	0.69 ^bc^ ± 0.02	0.68 ^bc^ ± 0.02	0.64 ^c^ ± 0.03	0.67 _d_ ± 0.03
	14 days	0.72 ^ab^ ± 0.03	0.67 ^bc^ ± 0.02	0.53 ^d^ ± 0.05	0.64 _e_ ± 0.09
	21 days	0.77 ^a^ ± 0.08	0.71 ^ab^ ± 0.02	0.57 ^d^ ± 0.07	0.68 _d_ ± 0.11
	Average	0.73 _a_ ± 0.06	0.69 _b_ ± 0.03	0.58 _c_ ± 0.07	0.66 ± 0.08
Main Effect (*p*-Value)					
	Laying Season (L)		Storage Time (ST)	L × ST	
Moisture	<0.0001		0.1310	0.0002	
Protein	0.3575		0.4716	0.0874	
Ash	<0.0001		0.0006	<0.0001	

_a–c_—Statistically significant differences (*p* ≤ 0.05) for the laying season are marked with different letters in the subscript; _d,e_—statistically significant differences (*p* ≤ 0.05) for the storage time are marked with different letters in the subscript; ^a–e^—statistically significant differences (*p* ≤ 0.05) for interaction type of laying season × storage time are marked with different letters in the superscript.

**Table 4:** Comparison of chemical composition of guinea fowl egg yolk during three laying seasons.

Item	Storage Time	Laying Season (Average ± SD)			
		I	II	III	Average
Moisture (%)	7 days	46.9 ^ab^ ± 1.9	46.1 ^b^ ± 0.8	48.9 ^ab^ ± 1.9	47.3 _d_ ± 2.0
	14 days	48.2 ^ab^ ± 3.1	47.9 ^ab^ ± 1.0	48.8 ^ab^ ± 2.7	48.3 _de_ ± 2.4
	21 days	48.7 ^ab^ ± 3.0	49.7 ^a^ ± 0.9	48.7 ^ab^ ± 1.1	49.0 _e_ ± 1.9
	Average	47.9 _a_ ± 2.7	47.9 _a_ ± 1.7	48.8 _a_ ± 1.9	48.2 ± 2.2
Protein (%)	7 days	16.5 ^a^ ± 0.6	16.7 ^a^ ± 0.6	17.0 ^a^ ± 0.4	16.8 _d_ ± 0.6
	14 days	16.3 ^a^ ± 0.6	16.6 ^a^ ± 0.4	16.3 ^a^ ± 0.7	16.4 _d_ ± 0.6
	21 days	16.8 ^a^ ± 1.0	16.5 ^a^ ± 0.4	16.3 ^a^ ± 0.7	16.5 _d_ ± 0.7
	Average	16.5 _a_ ± 0.8	16.6 _a_ ± 0.5	16.5 _a_ ± 0.7	16.6 ± 0.6
Fat (%)	7 days	31.1 ^ab^ ± 1.6	32.1 ^ab^ ± 1.4	31.6 ^ab^ ± 2.3	31.6 _d_ ± 1.8
	14 days	31.3 ^ab^ ± 1.4	31.8 ^ab^ ± 1.3	33.2 ^a^ ± 1.3	32.1 _d_ ± 1.5
	21 days	32.2 ^ab^ ± 1.2	30.6 ^b^ ± 1.5	33.3 ^a^ ± 1.6	32.0 _d_ ± 1.8
	Average	31.5 _b_ ± 1.4	31.5 _b_ ± 1.5	32.7 _a_ ± 1.9	31.9 ± 1.7
Ash (%)	7 days	1.98 ^a^ ± 0.09	1.68 ^c^ ± 0.11	1.74 ^bc^ ± 0.12	1.80 _d_ ± 0.17
	14 days	1.91 ^ab^ ± 0.13	1.68 ^c^ ± 0.08	1.62 ^c^ ± 0.24	1.74 _de_ ± 0.20
	21 days	1.88 ^ab^ ± 0.11	1.61 ^c^ ± 0.06	1.60 ^c^ ± 0.09	1.70 _e_ ± 0.16
	Average	1.93 _a_ ± 0.12	1.66 _b_ ± 0.09	1.65 _b_ ± 0.17	1.75 ± 0.18
Main Effect (*p*-Value)					
	Laying Season (L)		Storage Time (ST)	L × ST	
Moisture	0.1492		0.0045	0.0629	
Protein	0.8870		0.0753	0.0739	
Fat	0.0053		0.4320	0.0148	
Ash	<0.0001		0.0079	0.7691	

_a,b_—Statistically significant differences (*p* ≤ 0.05) for the laying season are marked with different letters in the subscript; _d,e_—statistically significant differences (*p* ≤ 0.05) for the storage time are marked with different letters in the subscript; ^a–c^—statistically significant differences (*p* ≤ 0.05) for interaction type of laying season × storage time are marked with different letters in the superscript.

**Table 5:** Trace element content (mg/kg wet tissue) in guinea fowl egg albumen.

Item	Storage Time	Laying Season (Average ± SD)			
		I	II	III	Average
Zn	7 days	0.17 ^a^ ± 0.09	0.19 ^a^ ± 0.09	0.10 ^a^ ± 0.05	0.15 _d_ ± 0.08
	14 days	0.12 ^a^ ± 0.06	0.16 ^a^ ± 0.09	0.09 ^a^ ± 0.02	0.13 _d_ ± 0.07
	21 days	0.14 ^a^ ± 0.09	0.16 ^a^ ± 0.10	0.08 ^a^ ± 0.03	0.14 _d_ ± 0.08
	Average	0.14 _a_ ± 0.08	0.17 _a_ ± 0.09	0.09 _b_ ± 0.03	0.14 ± 0.08
Si	7 days	3.21 ^b^ ± 0.52	3.35 ^ab^ ± 0.98	4.09 ^a^ ± 0.63	3.55 _d_ ± 0.81
	14 days	3.40 ^ab^ ± 1.06	3.47 ^ab^ ± 1.05	4.29 ^a^ ± 0.97	3.72 _d_ ± 1.07
	21 days	3.42 ^ab^ ± 0.98	2.78 ^b^ ± 0.49	4.57 ^a^ ± 1.02	3.59 _d_ ± 1.13
	Average	3.34 _b_ ± 0.86	3.20 _b_ ± 0.90	4.32 _a_ ± 0.88	3.62 _d_ ± 1.00
Fe	7 days	0.45 ^a^ ± 0.09	0.24 ^cd^ ± 0.04	0.19 ^d^ ± 0.06	0.29 _d_ ± 0.13
	14 days	0.33 ^bc^ ± 0.08	0.19 ^d^ ± 0.06	0.21 ^d^ ± 0.08	0.24 _e_ ± 0.10
	21 days	0.37 ^ab^ ± 0.12	0.19 ^d^ ± 0.06	0.22 ^d^ ± 0.05	0.26 _de_ ± 0.11
	Average	0.38 _a_ ± 0.11	0.21 _b_ ± 0.06	0.20 _b_ ± 0.07	0.26 ± 0.12
Cu	7 days	0.22 ^bc^ ± 0.03	0.23 ^bc^ ± 0.06	0.19 ^c^ ± 0.04	0.21 _e_ ± 0.04
	14 days	0.28 ^ab^ ± 0.03	0.22 ^c^ ± 0.02	0.25 ^abc^ ± 0.05	0.25 _d_ ± 0.04
	21 days	0.30 ^a^ ± 0.06	0.25 ^abc^ ± 0.04	0.22 ^c^ ± 0.02	0.26 _d_ ± 0.06
	Average	0.27 _a_ ± 0.05	0.23 _b_ ± 0.04	0.22 _b_ ± 0.04	0.24 ± 0.05
Ba	7 days	0.015 ^bc^ ± 0.005	0.019 ^ab^ ± 0.003	0.012 ^cd^ ± 0.004	0.015 _d_ ± 0.005
	14 days	0.009 ^d^ ± 0.003	0.016 ^bc^ ± 0.005	0.012 ^cd^ ± 0.004	0.012 _e_ ± 0.005
	21 days	0.022 ^a^ ± 0.004	0.011 ^cd^ ± 0.003	0.012 ^cd^ ± 0.004	0.015 _de_ ± 0.006
	Average	0.015 _a_ ± 0.006	0.015 _a_ ± 0.005	0.012 _b_ ± 0.004	0.014 ± 0.005
Sr	7 days	0.15 ^ab^ ± 0.02	0.14 ^ab^ ± 0.02	0.17 ^a^ ± 0.04	0.15 _d_ ± 0.03
	14 days	0.13 ^bc^ ± 0.02	0.12 ^bc^ ± 0.03	0.16 ^a^ ± 0.02	0.14 _e_ ± 0.02
	21 days	0.14 ^ab^ ± 0.03	0.10 ^c^ ± 0.02	0.16 ^a^ ± 0.02	0.13 _e_ ± 0.03
	Average	0.14 _b_ ± 0.02	0.12 _c_ ± 0.03	0.16 _a_ ± 0.03	0.14 ± 0.03
Mn	7 days	0.031 _a_ ± 0.005	0.022 ^bc^ ± 0.003	0.022 ^bc^ ± 0.003	0.025 _d_ ± 0.006
	14 days	0.023 ^bc^ ± 0.001	0.020 ^bc^ ± 0.002	0.019 ^c^ ± 0.002	0.021 _e_ ± 0.002
	21 days	0.025 ^b^ ± 0.006	0.021 ^bc^ ± 0.002	0.020 ^bc^ ± 0.002	0.022 _e_ ± 0.004
	Average	0.026 _a_ ± 0.006	0.021 _b_ ± 0.002	0.020 _b_ ± 0.003	0.022 ± 0.005
Al	7 days	0.28 ^a^ ± 0.04	0.18 ^b^ ± 0.04	0.15 ^b^ ± 0.05	0.21 _d_ ± 0.07
	14 days	0.27 ^a^ ± 0.06	0.15 ^b^ ± 0.02	0.14 ^b^ ± 0.05	0.19 _d_ ± 0.08
	21 days	0.27 ^a^ ± 0.07	0.12 ^b^ ± 0.02	0.17 ^b^ ± 0.05	0.19 _d_ ± 0.08
	Average	0.27 _a_ ± 0.06	0.15 _b_ ± 0.04	0.15 _b_ ± 0.05	0.19 ± 0.08
Se	7 days	0.11 ^a^ ± 0.01	0.11 ^a^ ± 0.02	0.11 ^a^ ± 0.01	0.11 _d_ ± 0.02
	14 days	0.11 ^a^ ± 0.01	0.12 ^a^ ± 0.01	0.12 ^a^ ± 0.01	0.11 _d_ ± 0.01
	21 days	0.11 ^a^ ± 0.01	0.13 ^a^ ± 0.01	0.11 ^a^ ± 0.01	0.12 _d_ ± 0.01
	Average	0.11 _b_ ± 0.01	0.12 _a_ ± 0.01	0.11 _ab_ ± 0.01	0.11 ± 0.01
Cr	7 days	0.34 ^a^ ± 0.08	0.34 ^a^ ± 0.05	0.33 ^a^ ± 0.05	0.34 _d_ ± 0.06
	14 days	0.35 ^a^ ± 0.04	0.33 ^a^ ± 0.05	0.33 ^a^ ± 0.03	0.33 _d_ ± 0.04
	21 days	0.32 ^a^ ± 0.06	0.37 ^a^ ± 0.02	0.36 ^a^ ± 0.06	0.35 _d_ ± 0.05
	Average	0.34 _a_ ± 0.06	0.35 _a_ ± 0.04	0.34 _a_ ± 0.05	0.34 ± 0.05
Pb	7 days	0.064 ^a^ ± 0.004	0.060 ^ab^ ± 0.005	0.059 ^ab^ ± 0.005	0.061 ^d^ ± 0.005
	14 days	0.057 ^b^ ± 0.002	0.058 ^ab^ ± 0.004	0.058 ^ab^ ± 0.004	0.058 _e_ ± 0.004
	21 days	0.059 ^ab^ ± 0.003	0.063 ^ab^ ± 0.004	0.060 ^ab^ ± 0.005	0.061 _d_ ± 0.004
	Average	0.060 _a_ ± 0.004	0.060 _a_ ± 0.004	0.059 _a_ ± 0.005	0.060 ± 0.004
Cd	7 days	0.0053 ^bc^ ± 0.0004	0.0057 ^ab^ ± 0.0005	0.0049 ^c^ ± 0.0003	0.0053 _d_ ± 0.0005
	14 days	0.0048 ^c^ ± 0.0004	0.0053 ^bc^ ± 0.0006	0.0048 ^c^ ± 0.0005	0.0050 _e_ ± 0.0005
	21 days	0.0060 ^a^ ± 0.0004	0.0053 ^bc^ ± 0.0003	0.0048 ^c^ ± 0.0004	0.0054 _d_ ± 0.0006
	Average	0.0054 _a_ ± 0.0006	0.0054 _a_ ± 0.0005	0.0048 _b_ ± 0.0004	0.0052 ± 0.0006
Main Effect (*p*-Value)					
	Laying Season (L)		Storage Time (ST)	L × ST	
Zn	0.0005		0.2710	0.9758	
Si	<0.0001		0.7303	0.3141	
Fe	<0.0001		0.0209	0.0351	
Cu	0.0001		0.0002	0.0065	
Ba	0.0005		0.0127	<0.0001	
Sr	<0.0001		0.0052	0.2629	
Mn	<0.0001		<0.0001	0.0328	
Al	<0.0001		0.2127	0.2135	
Se	0.0214		0.3411	0.4145	
Cr	0.8033		0.5600	0.2255	
Pb	0.4351		0.0068	0.0302	
Cd	<0.0001		0.0010	<0.0001	

_a–c_—Statistically significant differences (*p* ≤ 0.05) for the laying season are marked with different letters in the subscript; _d,e_—statistically significant differences (*p* ≤ 0.05) for the storage time are marked with different letters in the subscript; ^a–d^—statistically significant differences (*p* ≤ 0.05) for interaction type of laying season × storage time are marked with different letters in the superscript.

**Table 6:** Macronutrient content (mg/kg wet tissue) in guinea fowl egg albumen.

Item	Storage Time	Laying Season (Average ± SD)			
		I	II	III	Average
P	7 days	99 ^b^ ± 9	100 ^ab^ ± 6	101 ^ab^ ± 8	100 _d_ ± 7
	14 days	107 ^ab^ ± 6	101 ^ab^ ± 9	110 ^a^ ± 8	106 _e_ ± 8
	21 days	101 ^ab^ ± 8	109 ^ab^ ± 8	101 ^ab^ ± 5	104 _de_ ± 8
	Average	102 _a_ ± 8	103 _a_ ± 8	104 _a_ ± 8	103 ± 8
K	7 days	1380 ^a^ ± 113	1465 ^a^ ± 116	1443 ^a^ ± 122	1429 _d_ ± 119
	14 days	1448 ^a^ ± 85	1444 ^a^ ± 124	1437 ^a^ ± 50	1443 _d_ ± 88
	21 days	1494 ^a^ ± 126	1514 ^a^ ± 126	1472 ^a^ ± 127	1493 _d_ ± 123
	Average	1441 _a_ ± 116	1474 _a_ ± 122	1451 _a_ ± 103	1455 ± 113
Ca	7 days	52.9 ^a^ ± 9.0	53.1 ^a^ ± 6.1	51.0 ^ab^ ± 9.3	52.3 _d_ ± 8.0
	14 days	45.8 ^abc^ ± 6.2	38.2 ^c^ ± 4.7	42.3 ^bc^ ± 3.0	42.1 _e_ ± 5.6
	21 days	44.7 ^abc^ ± 8.9	36.9 ^c^ ± 4,3	41.5 ^c^ ± 5.0	41.0 _e_ ± 7.0
	Average	47.8 _a_ ± 8.7	42.7 _a_ ± 8.9	44.9 _ab_ ± 7.5	45.2 ± 8.6
Na	7 days	1545 ^de^ ± 38	1961 ^a^ ± 99	1848 ^b^ ± 45	1785 _d_ ± 190
	14 days	1503 ^e^ ± 31	1958 ^a^ ± 67	1749 ^c^ ± 64	1737 _e_ ± 197
	21 days	1849 ^b^ ± 28	1974 ^a^ ± 106	1638 ^d^ ± 85	1820 _d_ ± 161
	Average	1632 _c_ ± 160	1965 _a_ ± 90	1745 _b_ ± 108	1781 ± 185
Mg	7 days	114 ^a^ ± 10	115 ^a^ ± 5	120 ^a^ ± 9	116 _d_ ± 9
	14 days	117 ^a^ ± 8	114 ^a^ ± 13	123 ^a^ ± 13	118 _d_ ± 11
	21 days	116 ^a^ ± 11	114 ^a^ ± 10	115 ^a^ ± 11	115 _d_ ± 11
	Average	116 _a_ ± 9	114 _a_ ± 10	119 _a_ ± 11	116 ± 10
Main Effect (*p*-Value)					
	Laying Season (L)		Storage Time (ST)	L × ST	
P	0.6195		0.0097	0.0044	
K	0.4952		0.0734	0.6664	
Ca	0.0157		<0.0001	0.2749	
Na	<0.0001		<0.0001	<0.0001	
Mg	0.1784		0.4961	0.7936	

_a–c_—Statistically significant differences (*p* ≤ 0.05) for the laying season are marked with different letters in the subscript; _d,e_—statistically significant differences (*p* ≤ 0.05) for the storage time are marked with different letters in the subscript; ^a–e^—statistically significant differences (*p* ≤ 0.05) for interaction type of laying season × storage time are marked with different letters in the superscript.

**Table 7:** Trace element content (mg/kg wet tissue) in guinea fowl egg yolk.

Item	Storage Time	Laying Season (Average ± SD)			
		I	II	III	Average
Zn	7 days	43.5 ^abc^ ± 4.8	45.3 ^abc^ ± 2.8	44.0 ^abc^ ± 2.8	44.3 _f_ ± 3.5
	14 days	43.0 ^bc^ ± 3.9	46.1 ^abc^ ± 4.9	42.2 ^c^ ± 5.3	43.8 _f_ ± 4.9
	21 days	48.9 ^a^ ± 3.4	44.2 ^abc^ ± 3.6	48.1 ^ab^ ± 4.5	47.1 _d_ ± 4.3
	Average	45.1 _a_ ± 4.7	45.2 _a_ ± 3.8	44.8 _a_ ± 4.9	45.0 ± 4.4
Si	7 days	4.16 ^ab^ ± 0.83	5.01 ^a^ ± 1.26	4.86 ^a^ ± 0.93	4.68 _d_ ± 1.06
	14 days	4.20 ^ab^ ± 1.19	3.20 ^b^ ± 0.49	5.02 ^a^ ± 0.71	4.14 _d_ ± 1.12
	21 days	3.96 ^ab^ ± 1.40	2.81 ^b^ ± 0.29	5.45 ^a^ ± 1.76	4.08 _d_ ± 1.67
	Average	4.11 _b_ ± 1.13	3.67 _b_ ± 1.24	5.11 _a_ ± 1.21	4.30 ± 1.33
Fe	7 days	3.06 ^a^ ± 0.51	3.21 ^a^ ± 0.45	3.25 ^a^ ± 0.34	3.17 _d_ ± 0.43
	14 days	3.11 ^a^ ± 0.50	3.16 ^a^ ± 0.54	3.13 ^a^ ± 0.66	3.14 _d_ ± 0.55
	21 days	3.19 ^a^ ± 0.48	3.04 ^a^ ± 0.34	3.19 ^a^ ± 0.54	3.14 _d_ ± 0.45
	Average	3.12 _a_ ± 0.48	3.14 _a_ ± 0.44	3.19 _a_ ± 0.51	3.15 ± 0.48
Cu	7 days	2.08 ^a^ ± 0.21	2.13 ^a^ ± 0.22	2.06 ^a^ ± 0.22	2.09 _d_ ± 0.21
	14 days	2.01 ^a^ ± 0.14	2.16 ^a^ ± 0.24	1.98 ^a^ ± 0.24	2.05 _d_ ± 0.22
	21 days	2.03 ^a^ ± 0.09	2.09 ^a^ ± 0.16	1.98 ^a^ ± 0.17	2.04 _d_ ± 0.15
	Average	2.04 _a_ ± 0.15	2.13 _a_ ± 0.21	2.01 _a_ ± 0.21	2.06 ± 0.19
Ba	7 days	3.04 ^b^ ± 1.08	3.10 ^b^ ± 0.79	3.20 ^b^ ± 0.71	3.11 _e_ ± 0.85
	14 days	3.10 ^b^ ± 0.62	2.99 ^b^ ± 1.41	3.81 ^ab^ ± 0.83	3.30 _e_ ± 1.04
	21 days	3.96 ^ab^ ± 1.51	2.84 ^b^ ± 0.89	5.09 ^a^ ± 0.85	3.96 _d_ ± 1.43
	Average	3.37 _b_ ± 1.17	2.97 _b_ ± 1.03	4.03 _a_ ± 1.11	3.46 ± 1.18
Sr	7 days	1.94 ^abc^ ± 0.40	1.90 ^abc^ ± 0.30	2.17 ^ab^ ± 0.33	2.00 _d_ ± 0.36
	14 days	1.97 ^abc^ ± 0.29	1.71 ^c^ ± 0.37	2.23 ^ab^ ± 0.28	1.97 _d_ ± 0.37
	21 days	1.90 ^abc^ ± 0.33	1.82 ^b^ ± 0.22	2.27 ^a^ ± 0.20	2.00 _d_ ± 0.32
	Average	1.93 _b_ ± 0.33	1.81 _b_ ± 0.30	2.22 _a_ ± 0.27	1.99 ± 0.35
Mn	7 days	1.02 ^a^ ± 0.18	0.97 ^a^ ± 0.12	0.90 ^a^ ± 0.21	0.96 _d_ ± 0.18
	14 days	0.98 ^a^ ± 0.18	0.96 ^a^ ± 0.27	0.80 ^a^ ± 0.17	0.91 _d_ ± 0.22
	21 days	1.00 ^a^ ± 0.18	0.96 ^a^ ± 0.13	0.78 ^a^ ± 0.18	0.91 _d_ ± 0.19
	Average	1.00 _a_ ± 0.18	0.97 _a_ ± 0.18	0.83 _b_ ± 0.19	0.93 ± 0.19
Al	7 days	0.77 ^ab^ ± 0.11	0.73 ^ab^ ± 0.09	0.70 ^b^ ± 0.14	0.73 _d_ ± 0.11
	14 days	0.87 ^a^ ± 0.17	0.73 ^ab^ ± 0.15	0.72 ^ab^ ± 0.09	0.77 _d_ ± 0.15
	21 days	0.78 ^ab^ ± 0.05	0.68 ^b^ ± 0.12	0.76 ^ab^ ± 0.09	0.74 _d_ ± 0.10
	Average	0.81 _a_ ± 0.12	0.71 _b_ ± 0.12	0.73 _b_ ± 0.11	0.75 ± 0.12
Se	7 days	0.45 ^d^ ± 0.07	0.67 ^abc^ ± 0.07	0.62 ^bc^ ± 0.08	0.58 _d_ ± 0.12
	14 days	0.57 ^cd^ ± 0.09	0.80 ^a^ ± 0.10	0.58 ^c^ ± 0.07	0.65 _e_ ± 0.14
	21 days	0.60 ^c^ ± 0.10	0.75 ^ab^ ± 0.11	0.58 ^c^ ± 0.10	0.64 _e_ ± 0.13
	Average	0.54 _b_ ± 0.11	0.74 _a_ ± 0.10	0.59 _b_ ± 0.08	0.63 ± 0.13
Cr	7 days	0.37 ^a^ ± 0.03	0.37 ^a^ ± 0.05	0.35 ^a^ ± 0.01	0.36 _d_ ± 0.03
	14 days	0.36 ^a^ ± 0.02	0.37 ^a^ ± 0.04	0.36 ^a^ ± 0.02	0.36 ^d^ ± 0.03
	21 days	0.36 ^a^ ± 0.03	0.37 ^a^ ± 0.04	0.37 ^a^ ± 0.02	0.37 ^d^ ± 0.03
	Average	0.36 _a_ ± 0.03	0.37 _a_ ± 0.04	0.36 _a_ ± 0.03	0.37 ± 0.03
Pb	7 days	0.022 ^a^ ± 0.004	0.023 ^a^ ± 0.004	0.024 ^a^ ± 0.007	0.023 _d_ ± 0.005
	14 days	0.025 ^a^ ± 0.005	0.027 ^a^ ± 0.005	0.022 ^a^ ± 0.006	0.025 _d_ ± 0.005
	21 days	0.026 ^a^ ± 0.004	0.023 ^a^ ± 0.005	0.029 ^a^ ± 0.011	0.026 _d_ ± 0.007
	Average	0.024 _a_ ± 0.004	0.024 _a_ ± 0.005	0.025 _a_ ± 0.008	0.025 ± 0.006
Cd	7 days	0.0032 ^a^ ± 0.0002	0.0030 ^ab^ ± 0.0004	0.0023 ^c^ ± 0.0003	0.0028 _d_ ± 0.0005
	14 days	0.0033 ^a^ ± 0.0004	0.0030 ^ab^ ± 0.0004	0.0023 ^c^ ± 0.0006	0.0028 _d_ ± 0.0006
	21 days	0.0031 ^a^ ± 0.0003	0.0028 ^abc^ ± 0.0003	0.0026 ^bc^ ± 0.0003	0.0028 _d_ ± 0.0004
	Average	0.0032 _a_ ± 0.0003	0.0029 _b_ ± 0.0004	0.0024 _c_ ± 0.0004	0.0028 ± 0.0005
Main Effect (*p*-Value)					
	Laying Season (L)		Storage Time (ST)	L × ST	
Zn	0.9184		0.0050	0.0148	
Si	<0.0001		0.0655	0.0012	
Fe	0.8569		0.9545	0.8717	
Cu	0.0674		0.5145	0.8745	
Ba	0.0005		0.0041	0.0225	
Sr	<0.0001		0.8977	0.6570	
Mn	0.0011		0.4841	0.8918	
Al	0.0047		0.3244	0.2613	
Se	<0.0001		0.0075	0.0040	
Cr	0.2008		0.8639	0.8403	
Pb	0.8425		0.2440	0.0481	
Cd	<0.0001		0.9717	0.0966	

_a–c_—Statistically significant differences (*p* ≤ 0.05) for the laying season are marked with different letters in the subscript; _d–f_—statistically significant differences (*p* ≤ 0.05) for the storage time are marked with different letters in the subscript; ^a–d^—statistically significant differences (*p* ≤ 0.05) for interaction type of laying season × storage time are marked with different letters in the superscript.

**Table 8:** Macronutrient content (mg/kg wet tissue) in guinea fowl egg yolk.

Item	Storage Time	Laying Season (Average ± SD)			
		I	II	III	Average
P	7 days	7010 ^bc^ ± 364	7506 ^a^ ± 223	7181 ^ac^ ± 437	7232 _d_ ± 400
	14 days	6919 ^c^ ± 407	7560 ^a^ ± 294	7483 ^ab^ ± 391	7321 _d_ ± 459
	21 days	7125 ^ac^ ± 373	7396 ^ac^ ± 306	7465 ^ab^ ± 242	7329 _d_ ± 336
	Average	7018 _b_ ± 379	7487 _a_ ± 276	7377 _a_ ± 380	7294 ± 399
K	7 days	1261 ^ab^ ± 125	1266 ^ab^ ± 88	1336 ^ab^ ± 125	1288 _d_ ± 115
	14 days	1257 ^ab^ ± 145	1358 ^a^ ± 98	1286 ^ab^ ± 93	1300 _d_ ± 119
	21 days	1260 ^ab^ ± 88	1281 ^ab^ ± 154	1189 ^b^ ± 44	1244 _d_ ± 109
	Average	1259 _a_ ± 117	1302 _a_ ± 120	1271 _a_ ± 109	1277 ± 116
Ca	7 days	1933 ^a^ ± 243	2027 ^a^ ± 246	1975 ^a^ ± 176	1978 _d_ ± 220
	14 days	1917 ^a^ ± 179	2052 ^a^ ± 147	1924 ^a^ ± 260	1964 _d_ ± 204
	21 days	1964 ^a^ ± 177	2019 ^a^ ± 151	1944 ^a^ ± 188	1976 _d_ ± 170
	Average	1938 _a_ ± 196	2033 _a_ ± 181	1948 _a_ ± 205	1973 ± 197
Na	7 days	612 ^a^ ± 39	561 ^ab^ ± 19	590 ^ab^ ± 44	588 _d_ ± 40
	14 days	603 ^ab^ ± 36	558 ^b^ ± 20	468 ^c^ ± 31	543 _e_ ± 64
	21 days	588 ^ab^ ± 54	580 ^ab^ ± 45	474 ^c^ ± 14	547 _e_ ± 67
	Average	601 _a_ ± 44	567 _b_ ± 31	511 _c_ ± 65	559 ± 61
Mg	7 days	138 ^c^ ± 16	145 ^bc^ ± 16	155 ^ab^ ± 8	146 _de_ ± 15
	14 days	137 ^c^ ± 8	143 ^bc^ ± 9	148 ^abc^ ± 7	143 _e_ ± 9
	21 days	146 ^abc^ ± 10	145 ^bc^ ± 13	162 ^a^ ± 14	151 _d_ ± 14
	Average	140 _b_ ± 12	144 _b_ ± 12	155 _a_ ± 11	147 ± 13
Main Effect (*p*-Value)					
	Laying Season (L)		Storage Time (ST)	L × ST	
P	<0.0001		0.4882	0.1838	
K	0.3145		0.1213	0.0834	
Ca	0.1389		0.9586	0.9524	
Na	<0.0001		<0.0001	<0.0001	
Mg	<0.0001		0.0184	0.5060	

_a–c_—Statistically significant differences (*p* ≤ 0.05) for the laying season are marked with different letters in the subscript; _d,e_—statistically significant differences (*p* ≤ 0.05) for the storage time are marked with different letters in the subscript; ^a–c^—statistically significant differences (*p* ≤ 0.05) for interaction type of laying season × storage time are marked with different letters in the superscript.

**Table 9:** Fatty acids profile (%) in guinea fowl egg yolk.

Item	Storage Time	Laying Season (Average ± SD)			
		I	II	III	Average
Saturated fatty acids (SFA)					
C14:0	7 days	0.19 ^ab^ ± 0.02	0.20 ^a^ ± 0.02	0.18 ^ab^ ± 0.03	0.19 _d_ ± 0.02
	14 days	0.18 ^ab^ ± 0.03	0.20 ^a^ ± 0.01	0.17 ^ab^ ± 0.03	0.18 _d_ ± 0.03
	21 days	0.18 ^ab^ ± 0.02	0.19 ^ab^ ± 0.03	0.16 ^b^ ± 0.02	0.18 _d_ ± 0.03
	Average	0.18 _ab_ ± 0.02	0.20 _a_ ± 0.02	0.17 _b_ ± 0.03	0.18 ± 0.03
C16:0	7 days	12.0 ^ab^ ± 1.4	11.5 ^b^ ± 0.9	13.8 ^a^ ± 1.0	12.4 _d_ ± 1.5
	14 days	11.1 ^b^ ± 0.9	12.1 ^ab^ ± 0.9	11.6 ^ab^ ± 1.7	11.6 _de_ ± 1.3
	21 days	10.7 ^b^ ± 0.7	12.6 ^ab^ ± 1.4	10.8 ^b^ ± 1.4	11.3 _e_ ± 1.5
	Average	11.2 _a_ ± 1.1	12.0 _a_ ± 1.1	12.1 _a_ ± 1.9	11.8 ± 1.5
C17:0	7 days	0.14 ^ab^ ± 0.03	0.12 ^ab^ ± 0.02	0.11 ^b^ ± 0.02	0.12 _d_ ± 0.03
	14 days	0.13 ^ab^ ± 0.01	0.11 ^b^ ± 0.01	0.14 ^ab^ ± 0.03	0.13 _d_ ± 0.02
	21 days	0.14 ^ab^ ± 0.02	0.12 ^ab^ ± 0.02	0.16 ^a^ ± 0.03	0.14 _d_ ± 0.03
	Average	0.14 _a_ ± 0.02	0.12 _b_ ± 0.02	0.14 _a_ ± 0.03	0.13 ± 0.03
C18:0	7 days	8.54 ^ab^ ± 0.29	8.11 ^abc^ ± 0.52	8.36 ^ab^ ± 0.30	8.34 _d_ ± 0.41
	14 days	8.49 ^ab^ ± 0.91	7.61 ^bc^ ± 0.47	7.89 ^abc^ ± 0.55	8.00 _d_ ± 0.74
	21 days	7.95 ^abc^ ± 0.36	8.68 ^a^ ± 0.39	7.35 ^c^ ± 0.43	7.99 _d_ ± 0.67
	Average	8.33 _a_ ± 0.62	8.13 _ab_ ± 0.63	7.86 _b_ ± 0.59	8.11 ± 0.63
OTHER SFA	7 days	0.13 ^ab^ ± 0.03	0.12 ^abcd^ ± 0.01	0.084 ^d^ ± 0.009	0.11 _d_ ± 0.03
	14 days	0.12 ^ab^ ± 0.02	0.12 ^abc^ ± 0.01	0.10 ^bcd^ ± 0.01	0.11 _d_ ± 0.02
	21 days	0.15 ^a^ ± 0.03	0.087 ^cd^ ± 0.018	0.12 ^ab^ ± 0.01	0.12 _d_ ± 0.03
	Average	0.13 _a_ ± 0.03	0.11 _b_ ± 0.02	0.10 _b_ ± 0.02	0.11 ± 0.03
Monounsaturated fatty acids (MUFA)					
C16:1	7 days	2.13 ^ab^ ± 1.01	1.48 ^bc^ ± 0.39	1.46 ^bc^ ± 0.44	1.69 _d_ ± 0.71
	14 days	1.11 ^bc^ ± 0.44	1.97 ^ab^ ± 0.47	0.95 ^c^ ± 0.46	1.34 _d_ ± 0.63
	21 days	1.03 ^c^ ± 0.27	2.63 ^a^ ± 0.91	0.85 ^c^ ± 0.46	1.50 _d_ ± 1.00
	Average	1.42 _b_ ± 0.80	2.03 _a_ ± 0.77	1.09 _b_ ± 0.51	1.51 ± 0.80
C18:1 n-9c	7 days	48.5 ^ab^ ± 3.6	48.8 ^abc^ ± 2.3	49.7 ^a^ ± 2.1	49.0 _d_ ± 3.0
	14 days	48.1 ^abc^ ± 3.6	48.9 ^abc^ ± 1.5	48.4 ^bc^ ± 1.8	48.4 _e_ ± 2.6
	21 days	48.4 ^bc^ ± 2.8	47.7 ^ab^ ± 2.5	47.9 ^c^ ± 3.6	48.0 _e_ ± 3.7
	Average	48.33 _a_ ± 3.5	48.46 _a_ ± 2.1	48.80 _a_ ± 4.2	48.53 ± 3.4
C20:1	7 days	0.15 ^abc^ ± 0.01	0.15 ^abc^ ± 0.01	0.12 ^cde^ ± 0.01	0.14 _d_ ± 0.02
	14 days	0.13 ^bcd^ ± 0.03	0.17 ^a^ ± 0.01	0.11 ^e^ ± 0.02	0.14 _d_ ± 0.03
	21 days	0.15 ^ab^ ± 0.01	0.14 ^abcd^ ± 0.01	0.12 ^de^ ± 0.02	0.14 _d_ ± 0.02
	Average	0.14 _a_ ± 0.02	0.15 _a_ ± 0.02	0.11 _b_ ± 0.02	0.14 ± 0.02
OTHER MUFA	7 days	0.17 ^ab^ ± 0.03	0.14 ^bcd^ ± 0.02	0.14 ^bcd^ ± 0.02	0.15 _d_ ± 0.02
	14 days	0.13 ^bcd^ ± 0.03	0.15 ^abc^ ± 0.02	0.12 ^cd^ ± 0.02	0.13 _d_ ± 0.03
	21 days	0.13 ^bcd^ ± 0.02	0.19 ^a^ ± 0.03	0.10 ^d^ ± 0.02	0.14 _d_ ± 0.04
	Average	0.14 _b_ ± 0.03	0.16 _a_ ± 0.03	0.12 _c_ ± 0.02	0.14 ± 0.03
Polyunsaturated fatty acids (PUFA)					
C18:2 n-6c	7 days	26.2 ^bc^ ± 3.1	27.6 ^abc^ ± 1.8	24.7 ^c^ ± 1.8	26.2 _e_ ± 2.5
	14 days	28.5 ^abc^ ± 2.7	27.0 ^abc^ ± 1.4	29.1 ^ab^ ± 1.6	28.2 _d_ ± 2.1
	21 days	29.3 ^ab^ ± 1.9	25.9 ^bc^ ± 2.6	31.0 ^a^ ± 2.9	28.7 _d_ ± 3.2
	Average	28.0 _a_ ± 2.8	26.8 _a_ ± 2.0	28.3 _a_ ± 3.4	27.7 ± 2.8
C18:3 n-3	7 days	0.20 ^ab^ ± 0.04	0.16 ^abc^ ± 0.05	0.093 ^c^ ± 0.017	0.15 _e_ ± 0.06
	14 days	0.17 ^abc^ ± 0.05	0.17 ^ab^ ± 0.05	0.13 ^b^ ± 0.03	0.16 _de_ ± 0.04
	21 days	0.17 ^ab^ ± 0.03	0.21 ^a^ ± 0.03	0.18 ^ab^ ± 0.05	0.19 _d_ ± 0.04
	Average	0.18 _a_ ± 0.04	0.18 _a_ ± 0.05	0.13 _b_ ± 0.05	0.17 ± 0.03
C18:3 n-6	7 days	0.18 ^a^ ± 0.04	0.15 ^ab^ ± 0.05	0.080 ^b^ ± 0.016	0.14 _d_ ± 0.06
	14 days	0.15 ^ab^ ± 0.05	0.16 ^a^ ± 0.05	0.13 ^ab^ ± 0.04	0.15 _d_ ± 0.05
	21 days	0.16 ^ab^ ± 0.03	0.19 ^a^ ± 0.04	0.17 ^a^ ± 0.05	0.17 _d_ ± 0.04
	Average	0.16 _a_ ± 0.04	0.17 _a_ ± 0.05	0.12 _b_ ± 0.05	0.15 ± 0.05
C20:3 n-3	7 days	0.16 ^ab^ ± 0.01	0.14 ^abc^ ± 0.02	0.10 ^d^ ± 0.01	0.13 _e_ ± 0.03
	14 days	0.14 ^bc^ ± 0.02	0.14 ^abc^ ± 0.02	0.12 ^cd^ ± 0.01	0.13 _e_ ± 0.02
	21 days	0.14 ^abc^ ± 0.02	0.17 ^a^ ± 0.01	0.13 ^bc^ ± 0.02	0.15 _d_ ± 0.02
	Average	0.14 _a_ ± 0.02	0.15 _a_ ± 0.02	0.12 _b_ ± 0.02	0.14 ± 0.02
C22:6 n-3	7 days	1.16 ^ab^ ± 0.08	1.27 ^a^ ± 0.12	0.90 ^b^ ± 0.06	1.11 _d_ ± 0.18
	14 days	1.43 ^a^ ± 0.31	1.21 ^ab^ ± 0.18	0.91 ^b^ ± 0.19	1.18 _d_ ± 0.31
	21 days	1.27 ^a^ ± 0.21	1.15 ^ab^ ± 0.05	0.91 ^b^ ± 0.18	1.11 _d_ ± 0.22
	Average	1.29 _a_ ± 0.24	1.21 _a_ ± 0.13	0.91 _b_ ± 0.15	1.14 ± 0.24
OTHER PUFA	7 days	0.12 ^ab^ ± 0.05	0.11 ^ab^ ± 0.01	0.076 ^b^ ± 0.011	0.10 ± 0.03
	14 days	0.11 ^ab^ ± 0.02	0.11 ^ab^ ± 0.02	0.094 ^b^ ± 0.009	0.10 ± 0.02
	21 days	0.14 ^a^ ± 0.03	0.10 ^ab^ ± 0.02	0.11 ^ab^ ± 0.01	0.12 ± 0.03
	Average	0.12 _a_ ± 0.03	0.11 _ab_ ± 0.02	0.093 _b_ ± 0.016	0.11 ± 0.03
			Total percentages		
SFA	7 days	21.0 ^abc^ ± 1.3	20.0 ^bc^ ± 1.3	22.6 ^a^ ± 1.1	21.2 _d_ ± 1.6
	14 days	20.0 ^bc^ ± 1.7	20.1 ^abc^ ± 1.1	19.9 ^bc^ ± 1.3	20.0 _e_ ± 1.3
	21 days	19.1 ^c^ ± 1.0	21.6 ^ab^ ± 1.14	18.5 ^c^ ± 1.8	19.8 _e_ ± 1.9
	Average	20.0 _a_ ± 1.5	20.6 _a_ ± 1.4	20.3 _a_ ± 2.2	20.3 ± 1.7
MUFA	7 days	47.2 ^a^ ± 4.7	50.6 ^ab^ ± 2.5	51.4 ^a^ ± 2.4	51.0 _d_ ± 3.5
	14 days	49.5 ^ab^ ± 4.1	51.20 ^ab^ ± 1.9	49.60 ^ab^ ± 2.2	50.0 _e_ ± 3.2
	21 days	49.7 ^ab^ ± 3.0	50.7 ^a^ ± 3.4	46.30 ^b^ ± 4.1	49.8 _e_ ± 4.6
	Average	49.63 _a_ ± 4.2	50.80 _a_ ± 2.8	49.10 _a_ ± 4.6	50.26 ± 4.0
PUFA	7 days	28.0 ^bc^ ± 5.9	29.4 ^abc^ ± 3.6	26.0 ^c^ ± 3.4	30.0 _e_ ± 2.9
	14 days	27.8 ^abc^ ± 5.2	28.7 ^abc^ ± 3.0	30.5 ^ab^ ± 3.0	29.80 _d_ ± 4.0
	21 days	32.2 ^ab^ ± 3.8	27.7 ^bc^ ± 2.5	32.5 ^a^ ± 5.6	31.3 _d_ ± 6.4
	Average	29.6 _a_ ± 3.5	28.6 _a_ ± 3.0	29.66 _a_ ± 6.6	30.36 ± 5.5
n-6	7 days	26.5 ^bc^ ± 3.1	27.9 ^abc^ ± 1.9	24.9 ^c^ ± 1.8	26.4 _e_ ± 2.5
	14 days	28.8 ^abc^ ± 2.8	27.2 ^abc^ ± 1.5	29.3 ^ab^ ± 1.6	28.4 _e_ ± 2.1
	21 days	29.6 ^ab^ ± 2.0	26.1 ^bc^ ± 2.6	31.3 ^a^ ± 3.0	29.0 _d_ ± 3.3
	Average	28.3 _a_ ± 2.8	27.1 _a_ ± 2.0	28.5 _a_ ± 3.4	27.9 ±2.9
n-3	7 days	1.54 ^ab^ ± 0.08	1.59 ^a^ ± 0.14	1.11 ^c^ ± 0.06	1.41 _d_ ± 0.24
	14 days	1.75 ^a^ ± 0.29	1.55 ^ab^ ± 0.22	1.18 ^c^ ± 0.21	1.49 _d_ ± 0.33
	21 days	1.60 ^a^ ± 0.23	1.55 ^ab^ ± 0.07	1.24 ^bc^ ± 0.13	1.46 _d_ ± 0.22
	Average	1.63 _a_ ± 0.23	1.56 _a_ ± 0.15	1.18 _b_ ± 0.15	1.45 ± 0.27
			Ratios		
n-6/n-3	7 days	17.3 ^b^ ± 2.1	17.6 ^b^ ± 1.5	22.6 ^ab^ ± 2.4	19.2 _d_ ± 3.2
	14 days	16.9 ^b^ ± 3.8	17.8 ^b^ ± 1.8	25.7 ^a^ ± 5.8	20.1 _d_ ± 5.6
	21 days	18.8 ^b^ ± 2.7	17.0 ^b^ ± 2.1	25.6 ^a^ ± 4.7	20.5 _d_ ± 5.0
	Average	17.7 _b_ ± 2.9	17.5 _b_ ± 1.8	24.6 _a_ ± 4.5	19.9 ± 4.6
UFA/SFA	7 days	3.79 ^bc^ ± 0.32	4.01 ^abc^ ± 0.30	3.44 ^c^ ± 0.21	3.75 _e_ ± 0.36
	14 days	4.03 ^abc^ ± 0.45	3.99 ^abc^ ± 0.29	4.04 ^abc^ ± 0.31	4.02 _d_ ± 0.34
	21 days	4.24 ^ab^ ± 0.26	3.63 ^bc^ ± 0.26	4.44 ^a^ ± 0.52	4.10 _d_ ± 0.49
	Average	4.02 _a_ ± 0.38	3.88 _a_ ± 0.32	3.97 _a_ ± 0.55	3.96 ± 0.42
Main Effect (*p*-Value)					
	Laying Season (L)		Storage Time (ST)	L × ST	
C14:0	0.0021		0.2120	0.6975	
C16:0	0.0814		0.0252	0.0029	
C17:0	0.0208		0.1123	0.0307	
C18:0	0.0282		0.0699	0.0004	
OTHER SFA	<0.0001		0.4243	0.0001	
C16:1	0.0001		0.2150	0.0004	
C18:1 n-9c	0.1743		0.0035	0.0038	
C20:1	<0.0001		0.9144	0.0102	
OTHER MUFA	<0.0001		0.1631	0.0001	
C18:2 n-6c	0.1308		0.0042	0.0026	
C18:3 n-3	0.0012		0.0325	0.0155	
C18:3 n-6	0.0061		0.0620	0.0333	
C20:3 n-3	<0.0001		0.0157	0.0008	
C22:6 n-3	<0.0001		0.3397	0.1699	
OTHER PUFA	0.0019		0.1274	0.1684	
SFA	0.4506		0.0052	0.0002	
MUFA	0.0515		0.0075	0.0029	
PUFA	0.1026		0.0042	0.0007	
n-6	0.1475		0.0040	0.0026	
n-3	<0.0001		0.3765	0.3725	
n-6/n-3	<0.0001		0.4776	0.5200	
UFA/SFA	0.4357		0.0070	0.0003	

_a–c_—Statistically significant differences (*p* ≤ 0.05) for the laying season are marked with different letters in the subscript; _d,e_—statistically significant differences (*p* ≤ 0.05) for the laying season are marked with different letters in the subscript; ^a–e^—statistically significant differences (*p* ≤ 0.05) for interaction type of laying season × storage time are marked with different letters in the superscript; OTHER SFA = C6:0 + C8:0 + C10:0 + C11:0 + C12:0 + C13:0 + C15:0 + C20:0 + C21:0 + C22:0 + C24:0; OTHER MUFA = C14:1 + C15:1 + C17:1 + C18:1 n-9t + C22:1 n-9 + C24:1; OTHER PUFA = C20:2 n-6 + C20:3 n-6 + C20:4 n-6 + C20:5 n-3; UFA = MUFA + PUFA.

## Data Availability

The original contributions presented in the study are included in the article/Supplementary Materials, further inquiries can be directed to the corresponding author.

## Supplementary Materials

The following supporting information can be downloaded at: https://www.mdpi.com/article/10.3390/foods13132161/s1, Table S1: Feed composition for guinea fowl for the laying period in accordance with the recommendations. Ref. [55] is cited in the Supplementary Materials.
