# Supplementary material for: Evaluation of the Quality of Guinea Fowl (Numida meleagris) Eggs from Free-Range Farming Depending on the Storage Period and Age of Laying Hens
Source: Foods. 2024 Jul 8;13(13):2161. doi: 10.3390/foods13132161 (PMC11241129; doi:10.3390/foods13132161)
Supplement: Supplementary file 1 [file foods-13-02161-s001.zip › foods-3068991-supplementary.pdf]

Table S1. Feed composition for guinea fowl for the laying period per 100 kg in accordance with the recommendations [1].

Ingredients	%
Corn	30.0
Wheat	22.0
Soybean meal (CP 46%)	19.0
Barley	10.0
Fodder chalk	7.70
Soybean oil	4.00
Wheat bran	2.92
Fishmeal (CP 60%)	2.00
Monocalcium phosphate	1.30
Premix DJ-R	0.50
Sodium bicarbonate	0.30
NaCl	0.13
L-Lysine HCL	0.11
DL-Methionine	0.04

[1] Rutkowski A. (2021) [1].

References

1. Rutkowski, A. *Zalecenia Żywienia Perlic. W. Zalecenia Żywieniowe i Wartość Pokarmowa Pasz Dla Drobiu*; Smulikowska, R.S., Rutkowski, A., Eds.; Instytut Fizjologii i Żywienia Zwierząt: Jabłonna, Poland; Jana Kielanowskiego, Polski Oddział Światowego Stowarzyszenia Wiedzy Drobiarskiej PB WPSA Osielsko: Poznań, Poland, 2021; pp. 84–88. (In Polish)
